# CCL2 in Rheumatoid Arthritis: A Context-Dependent Cross-Cellular Node Serving as Biomarker and Therapeutic Target

**DOI:** 10.3390/cells15161461

**Published:** 2026-08-14

**Authors:** Bowen Shi, Ke Bai, Renping Liu, Nanzhen Kuang, Wei Cai

**Affiliations:** 1 The First Clinical Medical College, Nanchang University, Nanchang 330006, China; 4203122147@email.ncu.edu.cn; 2 The Second Clinical Medical College, Nanchang University, Nanchang 330006, China; 4203123456@email.ncu.edu.cn; 3 Department of Medical Microbiology & Immunology, School of Basic Medical Sciences, Jiangxi Medical College, Nanchang University, Nanchang 330031, China; renping66@ncu.edu.cn (R.L.); knz77@ncu.edu.cn (N.K.); 4 Department of Medical Genetics and Cell Biology, School of Basic Medical Sciences, Jiangxi Medical College, Nanchang University, Nanchang 330031, China

**Keywords:** monocyte chemoattractant protein-1, rheumatoid arthritis, synovial fibroblasts, inflammatory amplification network, CCL2/CCR2 axis

## Abstract

**Highlights:**

**What are the main findings?**

- CCL2 acts as a prominent cross-cellular node in rheumatoid arthritis, linking CCR2-dependent monocyte recruitment with synovial fibroblast activation, macrophage responses, and osteoclast-mediated bone remodeling.
- The effects of CCL2 vary with cellular source, receptor expression, disease stage, and the local synovial environment, indicating that it is a context-dependent component rather than the sole driver of RA inflammation.

**What are the implications of the main findings?**

- Direct CCL2/CCR2 blockades show preclinical activity but have not produced consistent clinical benefit. This gap indicates that future research should focus on network-wide combination therapies and patient-stratified regimens, instead of monotherapy targeting only the CCL2/CCR2 axis.
- Circulating CCL2 may complement established markers of preclinical risk, disease activity, remission, treatment response, and RA-related complications, but standardized assays and validated clinical cut-offs are still lacking.

**Abstract:**

C-C motif chemokine ligand 2 (CCL2) interacts with cytokines, adipokines, miRNAs, and multiple synovial cell populations. Experimental studies indicate that these interactions can form a CCL2-associated inflammatory amplification network across cell types. In cellular and animal models, increased CCL2 is associated with monocyte recruitment, synovial fibroblast activation, osteoclast-related bone remodelling, and vascular responses. Therapeutic strategies targeting the CCL2-centered inflammatory network include antagonists of the CCL2/CCR2 axis, natural products, synthetic compounds, conventional antirheumatic drugs, and emerging delivery-based approaches. Notably, direct CCL2/CCR2 inhibition has shown biological activity in experimental models but has not produced consistent clinical benefit in established rheumatoid arthritis (RA). Although these findings do not establish CCL2 as a dominant causal driver of RA, human observational studies suggest that circulating CCL2 may complement established markers in preclinical RA risk assessment, disease activity and remission classification, estimation of treatment response, and evaluation of RA-related complications such as interstitial lung disease. Of note, no validated concentration cut-off or standardized assay currently supports its routine clinical use. This review examines the CCL2-related inflammatory network in RA and evaluates its cellular mechanisms, therapeutic implications, and potential clinical applications.

## 1. Introduction

Rheumatoid arthritis (RA), an autoimmune disease, triggers chronic joint inflammation and pain through persistent synovial hyperplasia, cartilage degradation, and bone erosion [1,2]. According to an analysis based on the Global Burden of Disease Study 2021, the global age-standardized prevalence of RA was 208.90 per 100,000 population in 2021. Among the autoimmune diseases examined, RA ranked third after psoriasis and type 1 diabetes mellitus [3]. In China, the corresponding prevalence was 240.70 per 100,000 population. The global number of people living with RA is projected to reach approximately 31.7 million by 2050, imposing a substantial global health burden [4]. The synovial tissue (ST) in RA contains macrophages, dendritic cells, fibroblast-like synoviocytes (FLS), T cells, and B cells [5]. FLS derived from patients with RA (RA-FLS) exhibit enhanced migratory and invasive properties, thereby contributing substantially to joint destruction [6]. Given the multicellular nature of RA synovitis, this review examines CCL2 as one prominent cross-cellular mediator within a broader inflammatory network, rather than as a single upstream driver of RA pathogenesis. C-C motif chemokine ligand 2 (CCL2), also known as monocyte chemoattractant protein-1 (MCP-1), is a key member of the monocyte chemotactic protein family and binds with high affinity to C-C chemokine receptor type 2 (CCR2). Through this interaction, CCL2 induces chemotaxis and activates various immune cells. CCL2 recruits monocytes, T cells, mast cells, and basophils [7,8]. By binding to CCR2 on immune cells, CCL2 promotes monocyte recruitment and proinflammatory mediator production [9,10]. 

CCL2 levels are elevated in both the serum and synovial fluid of patients with RA [11,12], with prominent expression in macrophages and FLS [13]. Although RA synovitis involves multiple chemokine axes, CCL2 was selected as the focus of this review because it links CCR2-dependent monocyte recruitment with synovial fibroblast activation, macrophage responses, and osteoclast-mediated bone remodelling. Its involvement in both local inflammation and circulating biomarker studies also permits evaluation across mechanistic, therapeutic, and translational contexts. This review therefore focuses on CCL2, briefly compares it with other RA-related chemokines, and examines its cellular effects, therapeutic implications, and biomarker potential. This selection is further supported by quantitative clinical and molecular evidence. CCL2 was elevated approximately 3 years before symptom onset in ACPA-positive individuals, whereas high-sensitivity C-reactive protein, secretory phospholipase A2, and IL-6 did not show the same increase [14,15]. Moreover, serum CCL2 concentrations were significantly higher in patients with RA-associated interstitial lung disease (RA-ILD) than in those without RA-ILD (642.8 ± 268.6 vs. 406.9 ± 127.8 pg/mL), and were positively correlated with RA duration (r = 0.542) [16]. At the molecular level, α(1,2)-linked fucosylated CCL2 was increased in RA synovial tissue and synovial fluid, while inhibition of fucosyltransferase 1 reduced CCL2 levels, human microvascular endothelial cell migration, and tube formation [17]. Together, these findings further support the clinical and molecular relevance of CCL2 in RA.

## 2. A Positive Feedback Network: Molecular and Cellular Mechanisms Driving CCL2 Overexpression in RA

### 2.1. Convergent Upstream Regulation of CCL2

CCL2 expression in RA is regulated by numerous cytokines, adipokines, and non-coding RNAs. Despite their diversity, these regulators converge mainly on nuclear factor κB (NF-κB), mitogen-activated protein kinase (MAPK), phosphoinositide 3-kinase (PI3K)/AKT, and Janus kinase (JAK)/signal transducer and activator of transcription (STAT) signaling (Table 1). Cytokines can directly increase or suppress CCL2 expression; adipokines generally reinforce CCL2-associated inflammation and M1 macrophage polarization; and microRNA (miRNA) related circuits regulate CCL2 mainly by altering post-transcriptional repression [18,19,20]. IFN-γ and IL-4 exert cell- and context-dependent bidirectional effects on CCL2. Specifically, IFN-γ elevates CCL2 levels when co-present with IL-15, while it inhibits CCL2 expression without IL-15 co-stimulation. By contrast, IL-4 boosts baseline CCL2 secretion yet blocks the IL-15-triggered upregulation of CCL2 [18,21,22].

### 2.2. Cell-Specific Inflammatory Positive Feedback Network

**Figure 1:**
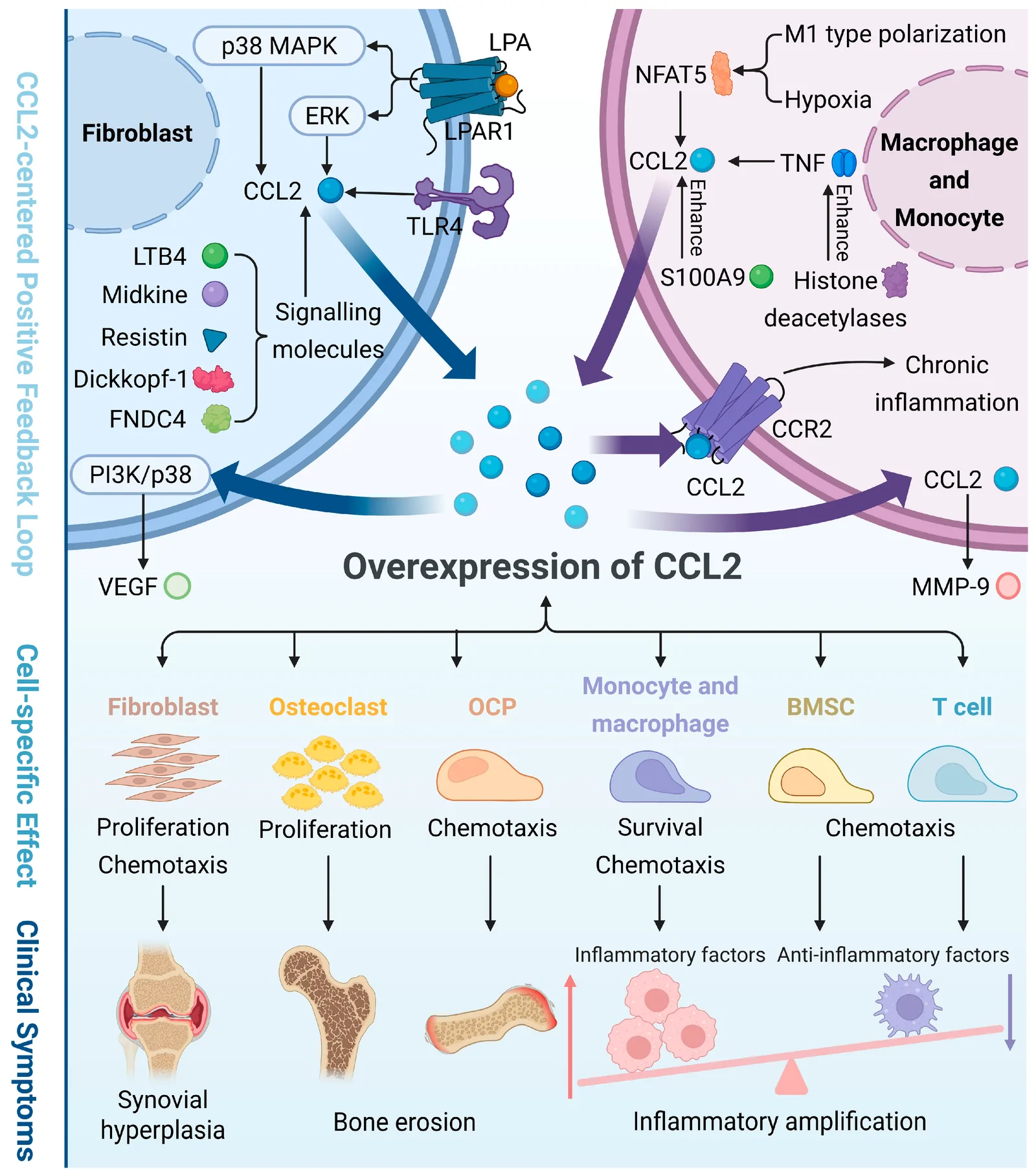
Cell-specific inflammatory positive feedback network. The figure can be divided into three panels. The increasing color intensity represents the progressive aggravation of the pathogenic process from the microscopic to the macroscopic level. Arrow indicates promotion effect. Top panel: CCL2-centered positive feedback loop. Fibroblasts, monocytes, and macrophages overexpress CCL2, thereby reinforcing CCL2 production through autocrine and paracrine loops. In fibroblasts, LPAR1 and TLR4 mediate pathways to upregulate CCL2 expression. Signals including LTB4, fibronectin type III domain-containing protein 4 (FNDC4), Dickkopf-1, midkine, and resistin regulate CCL2 expression. Elevated CCL2 stimulates fibroblasts to activate the PI3K/p38 pathway to release vascular endothelial growth factor (VEGF). In monocytes and macrophages, hypoxia and M1 macrophage polarization increase nuclear factor of activated T cells 5 (NFAT5) activity and CCL2 expression. S100A9 and histone deacetylases also enhance CCL2 expression. Increased CCL2 stimulates monocytes to release matrix metalloproteinase 9 (MMP-9), which will facilitate cartilage breakdown by activating RA-FLS. In addition, elevated CCL2 maintains an inflammatory state via CCR2. Middle panel: cell-specific effect induced by overexpressed CCL2. Bottom panel: Clinical symptoms of RA. CCL2 overexpression contributes to RA pathology through multiple cell types. FLS-derived CCL2 and VEGF promote synovial membrane hyperplasia; osteoclasts and osteoclast precursors (OCPs) contribute to bone erosion under the stimulation of CCL2; CCL2 damages synovial endothelial cells with an increasing risk of cardiovascular complications; CCL2 attracts Bone marrow mesenchymal stem cells (BMSCs), T cells, monocytes, and macrophages to amplify inflammation.

According to cell categories, we summarize the cell-specific inflammatory amplification network (Figure 1), especially in FLS, monocytes and macrophages.

#### 2.2.1. Synovial Fibroblasts

FLS are resident mesenchymal cells of the synovial joint [43]. Multiple upstream signals converge on CCL2 production in RA-FLS, but the supporting evidence varies in experimental context. Protein and receptor-associated regulators, including FNDC4, resistin, Dickkopf-1, midkine, immunoglobulin D Fc receptor, and TLR4, have been linked to altered CCL2 expression in cultured synovial fibroblasts or RA tissue [44,45,46,47,48,49]. Lipid-derived signaling provides another route: activation of the LPA–LPAR1 axis engages ERK1/2 and p38 MAPK signaling, whereas LTB4 increases CCL2 in experimental arthritis models [50,51,52]. Collectively, these studies suggest convergence on a limited number of intracellular pathways rather than independent CCL2-specific mechanisms. CCL2 plays a dual role in RA-FLS activation, acting through autocrine or paracrine pathways [53]. Studies using cultured RA-FLS showed that CCL2 promoted cell proliferation and migration [54]. Mixed-lineage leukemia 3, which is abundant in RA-FLS, increases H3K4me3 at the CCL2 promoter. This change activates NF-κB, increases proinflammatory factor expression, and reduces apoptosis [55]. CCL2 also induces vascular endothelial growth factor (VEGF) and CD31 via PI3K/p38, aiding synovial hyperplasia [56]. The principal pathways described above are summarized in the left panel of Figure 1.

#### 2.2.2. Monocytes and Macrophages

RELMα serves as a distinctive marker for M2 macrophages, which play a crucial role in wound healing [57]. In the early stages of antigen-induced arthritis (AIA), RELMα^+^ macrophages around blood vessels produce CCL2, which is linked to the buildup and transformation of monocytes into tissue-resident types like VSIG4^+^ macrophages [58]. As illustrated in the right panel of Figure 1, M1 polarization and hypoxia increase NFAT5 activity, which in turn enhances CCL2 expression and supports macrophage survival in RA [59]. Moreover, in monocyte or macrophage culture systems, S100A9 increased CCL2 secretion [60], while histone deacetylase activity was linked to CCL2 expression and osteoclast development [61].

CCR2 contributes to neutrophil accumulation in the synovium during acute arthritis [62,63]. Notably, CCL2 levels in synovial fluid (SF) do not correlate directly with monocyte migration [11], suggesting the involvement of additional factors. Macrophage CCR2 also promotes inflammation [62]. CCL2 stimulates monocytes to release MMP-9 [64], which subsequently promotes RA-FLS activity and cartilage degradation [65]. We also summarize the main pathways in the right panel of Figure 1.

#### 2.2.3. Osteoclasts, Osteoblasts and Chondrocytes

Bone erosion, a hallmark of RA, often presents as insidious and progressive joint pain and swelling, a process closely linked to the activation of osteoclasts and chondrocytes [66]. The number of OCPs increases in RA, and they express higher levels of CCR2, which correlates positively with CCL2 levels in vivo [67]. Osteoblasts can also continuously produce CCL2 in situ, possibly stimulated by IFN-γ. Notably, IL-6 significantly downregulates IFN-γ-induced CCL2 production [68]. Osteopontin (OPN), a bone-derived phosphoglycoprotein, plays a crucial role in bone remodeling [69]. Endogenous OPN production is increased by CCL2 and MIP-1β, and OPN can in turn induce CCL2 and MIP-1β expression in CD14^+^ cells, leading to leukocyte migration [70], thus creating a positive feedback loop centered on OPN and CCL2. During tumor necrosis factor (TNF) α-mediated osteoclast differentiation, CCL2 is also produced [71]. Furthermore, the number of chondrocytes is significantly reduced in RA, while apoptosis increases. These cells actively participate in bone erosion and sustained inflammation by secreting cytokines like CCL2 [72]. 

In osteoclast culture and experimental arthritis, CCL2 promoted osteoclast formation [73] and was associated with bone loss (Figure 1). Neutralizing antibodies against CCL2 reduced osteoclast formation by approximately 40% [71]. In arthritic mice, CCL2 expanded OCPs, particularly the CCR2hi subset, in periarticular tissues and the circulation, thereby promoting their joint homing and bone resorption [74] (Figure 1).

#### 2.2.4. Synovial Endothelial Cells

In endothelial cells, cytokines and cell-surface signaling molecules can induce CCL2 production. Macrophage migration inhibitory factor drives primary microvascular endothelial cells to secrete CCL2 [75]. CD45^+^ endothelial progenitor cells, prevalent in RA and linked to disease activity, also express high CCL2 [76]. Additionally, Tie-1, unique to endothelial cells, is overexpressed in RA and boosts CCL2 expression [77].

CCL2 can contribute to endothelial dysfunction. In CIA mice, CCL2 increases the expression of MCP-induced protein in vascular endothelial cells, contributing to aortic endothelial dysfunction [78] (Figure 1). As arthritis progresses, CCR2 expression in endothelial cells decreases and correlates inversely with the degree of inflammation [62], reflecting an adaptive response to reduce CCL2-mediated proinflammatory effects. However, CCL2 also exhibits anti-inflammatory properties. It recruits CD45^+^ endothelial progenitor cells, which can regulate macrophages via the SPI1/TGF-β signaling pathway to alleviate inflammation [76]. 

#### 2.2.5. Bone Marrow Mesenchymal Stem Cells (BMSCs)

BMSCs in the subchondral bone produce CCL2, and this production is further enhanced by the synergistic effects of TNF-α and IL-1β [79]. One mechanism involves citrullinated fibrinogen, a specific autoantigen in RA pathogenesis, which increases CCL2 by activating the TLR4-NF-κB pathway in BMSCs [80]. Stimulation with lipopolysaccharide (LPS) or TNF-α increases WNT5A expression threefold in BMSCs, leading to CCL2 production via activation of the NF-κB, MAPK, and Akt pathways [81]. By binding to the Gβγ subunit of the G-protein-coupled receptor complex, CCL2 activates PI3K/AKT, PAK, and ERK signaling pathways in BMSCs and induces their chemotactic migration [82] (Figure 1). In summary, these interactions may contribute to amplification of the local inflammatory network.

#### 2.2.6. T Cells

T cells do not primarily produce CCL2 but act as indirect regulators. For example, T-cell-derived microparticles can induce CCL2 secretion by FLS. However, T cells can also release CCL2 under certain conditions [83]. S100A8, a DAMP molecule, induces CCL2 secretion in Jurkat T cells [84], though its effect on normal T cell subsets remains unconfirmed.

CCL2 plays a key role in the migration of CCR2^+^ T cells [85], with higher CCR2 levels linked to stronger migration [86] (Figure 1). In RA patients’ ST, Th1 cells dominate T cell populations. In migration assays using T cells from RA synovial tissue, CCL2 blockade reduced Th1-cell migration by 56–77% [87]. Moreover, BMSC-derived CCL2 can inhibit the differentiation of CD4^+^ T cells into Th17 cells [88], suggesting a potential anti-inflammatory effect.

### 2.3. CCL2 Within the Broader Chemokine Network in RA

RA synovitis is coordinated by several chemokine axes rather than CCL2 alone. CXCL8 primarily promotes neutrophil recruitment and activation within the synovial compartment, whereas CXCL10/CXCR3 is more closely associated with interferon-driven recruitment of CXCR3-positive T cells [89,90]. CCL5 contributes to T-cell and monocyte trafficking and, together with CCL2 and CXCL12, supports Th1-cell migration toward RA synovial tissue [87]. By contrast, CXCL13/CXCR5 is linked to B-cell accumulation and the organization of ectopic lymphoid follicles [91]. These chemokines therefore emphasize different cellular and pathological compartments within RA synovitis.

CCL2 is neither the only nor a universally dominant chemokine in RA. Its relevance lies in its participation in cross-cellular positive-feedback loops linking CCR2-dependent monocyte recruitment with synovial fibroblast activation, macrophage responses, osteoclast-lineage expansion, endothelial dysfunction, and BMSC trafficking. Through these reciprocal interactions, increased CCL2 not only links leukocyte trafficking with stromal activation and bone remodeling but also amplifies and sustains synovial inflammation [74,92,93,94]. Prospective pre-diagnostic human studies also indicate potential clinical relevance. Circulating CCL2 was elevated approximately 3 years before symptom onset in ACPA-positive individuals, whereas high-sensitivity CRP, secretory phospholipase A2, and IL-6 were not consistently elevated at the same stage [14,15]. However, CCL2 remains part of a redundant chemokine network, and overlapping CCR2-, CCR5-, and other chemokine-receptor pathways may preserve leukocyte recruitment when a single axis is inhibited [95]. CCL2 should therefore be regarded as a prominent but non-exclusive amplifier within the RA chemokine network.

## 3. Therapeutic Applications

**Figure 2:**
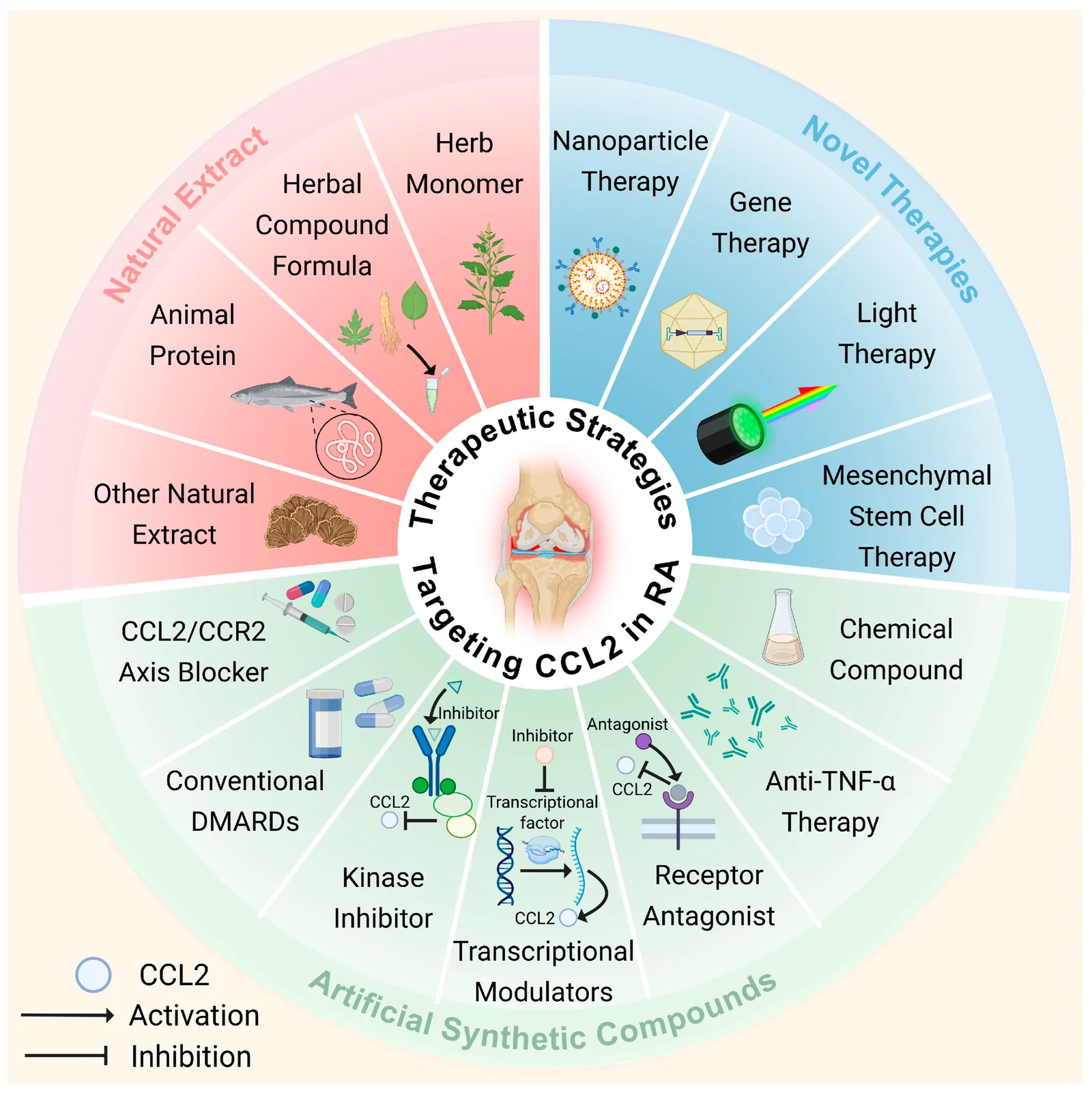
Therapeutic strategies targeting CCL2 in RA. It can be divided into natural extracts, artificial synthetic compounds, and novel therapies. DMARDs: disease-modifying antirheumatic drugs.

Therapeutic agents include natural extracts, artificial compounds, and novel therapies (Figure 2). The interventions reviewed in this section differ substantially in evidence level and translational maturity. Most natural products, synthetic compounds, and delivery-based approaches have been evaluated primarily in vitro or in experimental arthritis models. Therefore, changes in CCL2 should be interpreted as mechanistic or pharmacodynamic observations rather than evidence of therapeutic efficacy in established human RA. Importantly, direct CCL2/CCR2 blockade has not demonstrated consistent clinical benefit in randomized studies [10,96]. The reasons for this preclinical–clinical gap, including chemokine-network redundancy, disease stage, and target-engagement limitations, are discussed further in Section 5.

### 3.1. Natural Extract

#### 3.1.1. Herb Monomer

The natural products in this subsection have been evaluated mainly in cultured RA-FLS or experimental arthritis. Representative monomers, including celastrol, magnoflorine, quercetin, and Ganoderma lucidum polysaccharide peptide, reduced CCL2-associated inflammation mainly through inhibition of NF-κB and MAPK-related signaling [97,98,99,100,101,102]. Other compounds acted through AMPK/mTOR/ULK1, TLR4, macrophage polarization, or related inflammatory pathways, but the evidence generally came from individual preclinical studies, and CCL2 was often one of several downstream outcomes [103,104,105]. The remaining compounds and their principal limitations are summarized in Table 2. 

#### 3.1.2. Herb Formula

Herbal formulas regulate CCL2-associated inflammation mainly through NF-κB inhibition, PPARγ activation, and macrophage polarization, with multicomponent preparations acting through several pathways (Table 2).

Wutou decoction modulates macrophage polarization and reduces CCL2 expression [106]. Qing-Luo-Yin reduces CCL2 while increasing PPAR-γ expression [107]. Heiguteng Zhuifenghuoluo capsule decreases CCL2 by downregulating NF-κBp65, p-NF-κBp65, and RANKL [108]. 

Other polyherbal preparations include Majoon ushba, a multicomponent herbal formulation [109], which inhibits NF-κB-p65 expression and phosphorylation and is associated with marked reductions in IL-6 and CCL2 [43]. Triphala, a traditional Ayurvedic formulation containing Terminalia chebula, Terminalia bellerica, and Emblica officinalis, reduces CCL2 and TNF-α [110], and inhibits the nuclear translocation of p-NF-κBp65, thereby reducing COX-2 expression and alleviating AIA symptoms [111]. Trikatu reduced CCL2 expression in RA-FLS and reduced arthritis severity in the experimental model [112]. The combination of curcumin with vitamin D3 and omega-3 fatty acids produced a more pronounced effect than curcumin alone and improved outcomes in CIA [113].

#### 3.1.3. Animal Protein and Other Natural Extracts

Animal-derived products and other natural extracts mainly reduce CCL2-associated inflammation through TLR4/MyD88–NF-κB inhibition or decreased inflammatory-cell infiltration.

Sturgeon cartilage’s type II collagen lowers CCL2 via the TLR4/MyD88-NF-κB pathway, easing RA [114]. Salmon cartilage proteoglycan reduces CCL2, improving CIA [115]. In addition to the substances mentioned above, processed products from natural ingredients also play a similar role. Oyster mushroom β-glucan reduced proinflammatory cytokine levels and showed anti-inflammatory effects in a rabbit RA model [116]. Additionally, sesame and rice bran oils’ components inhibit paw inflammation severity and oxidative stress in RA models [117].

We summarize the mechanisms and evidence level of natural extracts listed above (Table 2).

**Table 2:** Categories and mechanisms of natural products targeting CCL2 in RA. All studies summarized in this table were conducted in animal models. All evidence remains preclinical, and none of the cited studies establishes routine clinical efficacy in patients with RA.

Subcategory	Compound	Mechanism	Main Limitation	References
Herb Monomer	Celastrol	Inhibition of the NF-κB pathway, RA-FLS proliferation and invasion	No clinical validation; broad pathway effects	[98]
	Quercetin	Inhibition of NF-κB pathway; downregulation of TNF-α	No clinical validation; separate experimental systems	[100,118]
	Magnoflorine	Inhibition of NF-κB and MAPK pathways	Single preclinical study; no clinical validation	[99]
	Ginkgolide B	Regulation of Wnt5a/JNK/NF-κB axis	Single animal study; CCL2 is one of several outcomes	[119]
	Nobiletin	Inhibition of NF-κB pathway	Single animal study; no human RA validation	[120]
	Withaferin-A	Inducing M2-type polarization of macrophages	Formulation-specific; no human data	[105]
	Eupalinolide B	Regulation of AMPK/MTOR/ULK-1 pathway	Recent single-study evidence; independent replication lacking	[103]
	Saponin 1	Downregulation of COX-2 and CCL2; shifting the immune response toward Th2-type	Single animal study; CCL2 is a secondary outcome	[121]
	Damsin or neoambrosin	Regulation of Akt/ERK1/2/STAT3 pathway	Effects of the individual compounds were not fully separated	[122]
	Q-1 (a compound extracted from the Miao medicine tiekuaizi)	Inhibition of NF-κB pathway	Single animal study; pharmacokinetic evidence is limited	[123]
	Cannabidiol extract	Inhibition of NF-κB pathway	Extract composition and dose standardization remain uncertain	[124]
	Adlay seed extract	Inhibition of pro-inflammatory factor production; alleviation of oxidative stress	Active constituents and direct CCL2 target are unclear	[125]
	Xuelian injection	Inhibition of TLR4 signaling pathways	Multicomponent preparation; active component is unclear	[104]
Herbal Compound Formula	Combination of curcumin, vitamin D3 and omega-3 combination	Suppression of serum anti-collagen antibodies; inhibition of cellular infiltration; downregulation of MMP	Combination effect cannot be attributed to one component or to CCL2	[113]
	Triphala	Inhibition of pNF-κBp65 from moving into the nucleus; downregulation of COX-2 expression	Multicomponent formula; active constituent is unclear	[111]
	Qing-luo-yin	Activation of PPARγ expression	Multicomponent formula; no human RA validation	[107]
	Wutou decoction	Activation of PPARγ expression; inducing M2-type polarization of macrophages	Multicomponent formula; CCL2-specific contribution is unclear	[106]
	Heiguteng zhuifenghuoluo capsule	Inhibition of NF-κB pathway	Single animal study; active constituents are unclear	[108]
	Majoon ushba	Inhibition of NF-κB pathway	Multicomponent formula; no human validation	[109]
	Trikatu	Inhibition of NF-κB pathway	Multicomponent formula; evidence remains preclinical	[112]
Animal Protein	Sturgeon cartilage type II collagen	TLR4/MyD88-NF-κB	Animal evidence only; clinical translation unclear	[114]
	Salmon cartilage proteoglycan	Inhibition of macrophages, neutrophils, and osteoclasts infiltration	Animal evidence only; direct CCL2 mechanism is unclear	[115]
Other Natural Extract	Extract from oyster mushroom (β-(1,3/1,6)-D-glucan)	Downregulation of CCL2	CCL2 is a secondary outcome; no human validation	[116]
	Minor components and fatty acids from sesame oil and rice bran oil	Inhibition of hydrolytic enzymes (collagenase, elastase, and hyaluronidase) in the synovial tissue	Active component and direct CCL2 mechanism are unclear	[117]

### 3.2. Artificial Synthetic Compounds

#### 3.2.1. CCL2/CCR2 Axis-Targeting Drugs

Direct blockade of the CCL2/CCR2 axis has produced inconsistent results across experimental and clinical settings. In a randomized phase II trial, the anti-CCL2 antibody ABN912 did not improve clinical or synovial outcomes in patients with RA and was accompanied by a sustained increase in circulating total CCL2, consistent with the formation of antibody–CCL2 complexes [96]. By contrast, preclinical studies showed that CCR2 antagonism reduced inflammatory mediator release in cultured RA-FLS and alleviated arthritis in CIA mice [126], while preventive blockade of the CCL2/CCR2 axis suppressed osteoclast activity and bone resorption in another mouse arthritis model [74]. These findings support the biological relevance of the pathway but do not establish clinical efficacy.

#### 3.2.2. Conventional Disease-Modifying Antirheumatic Drugs (DMARDs)

Conventional DMARDs such as methotrexate (MTX) reduce CCL2 production by synovial monocytes, whereas anakinra does not show the same effect [127]. MTX may disrupt gut microbiota homeostasis; however, co-administration with Lactiplantibacillus plantarum LS/07 reduced CCL2 production in AIA [128]. In experimental models, MTX combined with carnosic acid or N-feruloylserotonin reduced IL-17A and CCL2 and attenuated hind-paw swelling [129,130]. Leflunomide, an alternative for patients who do not respond to or tolerate MTX, also reduced CCL2 during RA treatment [131]. In AIA rats, galantamine reduced CCL2 levels in a dose-dependent manner and showed greater effects than leflunomide in the cited study [132].

#### 3.2.3. Kinase Inhibitors and Transcriptional Modulators

Several agents in this category have been used clinically or evaluated as therapies for RA. For example, both Peficitinib and Baricitinib are drugs approved by the Food and Drug Administration [133]. Peficitinib may suppress CCL2 secretion, monocyte migration, and RA-FLS proliferation, potentially through STAT1, STAT3, and STAT5 [134]. Baricitinib, by blocking JAK signaling from oncostatin M in osteoarthritic FLS, reduces IL-6 and CCL2 [135]. Additionally, Iguratimod showed clinical efficacy in a 28-week multicenter, randomized, double-blind phase III trial in patients with active RA [136]. Its inhibition of JNK/p38 signaling, CCL2 expression, and RA-FLS proliferation, migration, and invasion was demonstrated separately in vitro [137]. The JAK3 inhibitor PF-956980 also reduced CCL2, although the pan-JAK inhibitors CP-690,550 and INCB028050 produced stronger suppression of CCL2 in RA-FLS [138]. Bruton’s tyrosine kinase (BTK) inhibitor PCI-32765 also reduces CCL2 and limits monocyte and macrophage infiltration in CIA mice [139]. CX-4945, a casein kinase 2 inhibitor, reduces CCL2 expression and alleviates arthritis severity in CIA mice [140]. 

Transcriptional modulators like KRN2, an NFAT5 inhibitor, curb CCL2 and RA-FLS migration [6]. Another NFAT5 inhibitor, KRN5, is orally administered and metabolically stable and showed greater efficacy than MTX in CIA mice [141]. 

#### 3.2.4. Receptor-Targeting Therapies

All-trans retinoic acid reduces CCL2 expression and improves arthritis [142]. Although cannabinoid receptor 2 (CB2) is elevated in the RA synovium, JWH133, a CB2 agonist, can further inhibit TNF-α-induced CCL2 production [143]. Another CB2 agonist, JWH-015, reduces CCR2 expression on monocytes and inhibits their migration towards CCL2 [144]. The TLR4 pathway is pivotal in inflammation. Compound 32, a G-protein receptor (GPR) 183 antagonist, cuts CCL2 expression and improves RA [145]. 

#### 3.2.5. Anti-TNF Therapy

Infliximab can lower CCR2 expression on T cells [146], hinder Ly6C^+^ macrophage apoptosis and monocyte migration [147]. When used with MTX, anti-TNF-α antibody therapy swiftly improves clinical symptoms and keeps chemokine levels low [148]. It also alters monocyte subsets, increasing non-classical and reducing classical monocytes [149]. Anti-CD11b monoclonal antibody affects CCL2 and osteoprotegerin, reducing joint inflammation [150]. An anti-TWEAK monoclonal antibody similarly reduced CCL2 expression and alleviated CIA [151].

#### 3.2.6. Chemical Compound

p-Coumaric acid suppresses CCL2 expression in rat ankle joints and serum [152]. BT2, a dibenzoxazepine compound, inhibits monocyte migration towards CCL2 by blocking ERK phosphorylation [153]. A TNF-α inhibitor from a 3-methylcinnamamide-lysine mimetic also cuts CCL2 secretion [154]. Relevant information on artificial synthetic compounds is listed in Table 3. 

### 3.3. Novel Therapies

Novel approaches investigated in experimental RA include cell-based, light-based, gene-delivery, and nanoparticle-based strategies. In arthritic mice, combining mesenchymal stem cells with IL-4 reduced CCL2 by 68% and improved disease outcomes [162]. Yet, bone marrow-derived cells’ effect on CCL2 is unclear [163], warranting more investigation.

As for light therapy, both light-emitting diode (LED) irradiation and low-level laser irradiation exert anti-inflammatory effects by reducing CCL2 [164,165]. In terms of gene therapy, SIRT6 (silent information regulator 6) is downregulated in RA, whereas adenoviral SIRT6 delivery inhibits CCL2 expression through the MyD88–ERK pathway [166].

Several nanoparticle-based approaches have been evaluated in experimental arthritis. Piroxicam formulated as nanoparticles enables intra-articular targeted delivery, reduces CCL2 expression, and alleviates inflammation [167]. Selenium nanoparticles reduced CCL2 levels in the cited animal model [168]. Chitosan nanoparticles loaded with eugenol inhibited CCL2 expression in experimental arthritis [169]. Chitosan nanoparticles encapsulating MeuKTx, a potassium channel inhibitory peptide targeting Kv1.3, significantly reduced TGF-β and CCL2 [170]. 

Table 4 summarizes these novel therapies. To avoid excessive detail, Table 1, Table 2, Table 3 and Table 4 do not include in vitro studies; the corresponding in vitro therapeutic studies are summarized in Supplementary Table S1.

Overall, the therapeutic strategies reviewed above modulate CCL2 either directly through the CCL2/CCR2 axis or indirectly through broader inflammatory pathways. Most of the available evidence remains preclinical, and no CCL2- or CCR2-directed strategy is currently used in routine RA treatment.

## 4. Biomarkers for Prediction, Disease Activity and Prognostic Monitoring

Circulating CCL2 has been investigated across several clinically relevant stages of RA, including preclinical risk, established disease activity, treatment response, longitudinal monitoring, and RA-related complications. The available human studies indicate that its potential value differs according to disease stage and clinical context. In individuals who subsequently developed RA, circulating CCL2 was elevated up to 3 years before symptom onset, with the clearest increase observed among anti-citrullinated protein antibody (ACPA) positive individuals, whereas high-sensitivity CRP, secretory phospholipase A2, and IL-6 were not consistently elevated at the same stage [14,15]. This temporal pattern suggests that CCL2 may reflect an early chemokine-related process within an autoantibody-positive subgroup and may complement conventional acute-phase markers. Multimarker models incorporating CCL2 with CRP, IL-6, CXCL8, or TNF-α may further support preclinical risk stratification. CCL2 has also been examined as a candidate marker for RA-related complications. In one cohort, combined measurement of CCL2, vascular cell adhesion molecule-1 (VCAM-1), and asymmetric dimethylarginine showed discriminatory value for RA-associated interstitial lung disease (RA-ILD) [16]. Higher CCL2 was also associated with cardiovascular risk in the presence of a broader inflammatory profile [171]. 

Circulating CCL2 has been associated with established measures of RA disease activity, including the disease activity score in 28 joints (DAS28), and CCL2/MCP-1 has been incorporated into an experimental DAS28-CCL2 score [172]. DAS28-CCL2 is calculated as follows:

DAS28-CCL2 = 0.56 × √TJC28 + 0.28 × √SJC28 + 0.39 × ln[CCL2 (pg/mL)] + 0.014 × PGA (mm)

In this formula, TJC28 and SJC28 denote the tender and swollen joint counts among 28 joints, respectively. The term ln denotes the natural logarithm, circulating CCL2 is expressed in pg/mL, and PGA denotes the patient global assessment measured on a 0–100 mm visual analogue scale [172,173]. In one prospective cohort, a DAS28-CCL2 cut-off of <2.2 showed a sensitivity of 91.18% and specificity of 97.39% for modified American Rheumatism Association remission, and a sensitivity of 69.07% and specificity of 97.97% for 2011 American College of Rheumatology/European Alliance of Associations for Rheumatology remission [173]. These results reflect the performance of DAS28-CCL2 in classifying disease activity and remission status rather than in diagnosing RA. 

Compared with DAS28-ESR and DAS28-CRP, the potential additional value of DAS28-CCL2 may lie in the distinct biological information provided by its laboratory component. Erythrocyte sedimentation rate (ESR) and CRP predominantly reflect the systemic acute-phase response, whereas CCL2 is involved in chemokine-mediated monocyte recruitment and can be produced by activated monocytes and fibroblasts at inflammatory sites [54,173]. DAS28-CCL2 may therefore complement conventional disease-activity indices by capturing a chemokine-related component of synovial inflammation. In the reported prospective cohort, the DAS28-CCL2 remission cut-off showed high specificity for stringent remission definitions and better agreement with these definitions than the conventional DAS28-ESR remission cut-off [173]. However, these findings were derived from a limited number of cohorts, mainly from the same research group. Standardized CCL2 assays, independently validated cut-off values, and validation across different populations and treatment regimens remain lacking. 

This degree of validation contrasts with that available for conventional acute-phase measures. DAS28-ESR and DAS28-CRP have been evaluated in multiple independent RA populations, including a large Japanese observational cohort, early and established RA cohorts, and clinical-trial populations [174,175,176]. These studies nevertheless show that ESR- and CRP-based assessments are not completely interchangeable and may yield different disease-activity classifications or cut-off values. In addition, age, sex, and BMI can differentially influence ESR and CRP in patients with RA [177]. Thus, the advantage of CRP and ESR is not that they are free from biological variability, but that their performance and limitations have been characterized across substantially larger and more diverse clinical populations. Comparable cross-population validation is not yet available for circulating CCL2.

Therefore, DAS28-CCL2 should currently be considered an investigational complementary index rather than a replacement for DAS28-ESR or DAS28-CRP. In a cohort of patients with untreated early RA, baseline chemokine levels, including CCL2, did not distinguish patients who subsequently achieved clinical remission at week 24 from those who did not [178]. This finding indicates that circulating CCL2 alone has limited value as a pretreatment predictor of short-term remission and may be more informative when incorporated into a composite disease-activity or multimarker treatment-stratification model. The favorable remission-classification performance of DAS28-CCL2 contrasts with the lack of pretreatment predictive value for circulating CCL2 in an independent early-RA cohort. Together, these findings suggest that the clinical performance of CCL2 may depend on disease stage, population, and intended application rather than represent a universally reproducible biomarker signal [173,178]. Longitudinal studies further indicate that circulating CCL2 changes dynamically during RA treatment. During Leflunomide therapy, serum CCL2 was measured at baseline and after 3, 6, 9, and 12 months; a significant reduction was evident by month 3 and persisted through month 12 [131]. Etanercept-based studies also reported reduced serum CCL2 at 3 and 6 months, while another cohort detected a significant decrease by month 3 during serial follow-up to 12 months [179,180]. In women receiving TNF inhibitors combined with MTX, serum CCL2 was assessed at baseline and after 3, 9, and 15 months and was most clearly reduced at month 15, approaching the level observed in healthy controls [181]. However, treatment-specific differences were observed: the reduction was significant in the Adalimumab subgroup but not in the Etanercept subgroup. Consistent with this heterogeneity, a separate one-year Etanercept study reported increased serum CCL2 despite improvements in DAS28, CRP, and ESR, with the increase associated with body weight and body mass index (BMI) [157]. These findings suggest that early and intermediate reductions in CCL2 may accompany suppression of inflammatory activity, whereas longer-term changes depend on the therapeutic agent and metabolic context.

Compared with CRP and ESR, which mainly reflect the systemic acute-phase response, CCL2 may provide complementary information on chemokine-driven monocyte recruitment and synovial inflammatory activity. Its potential additional value may therefore lie in multimarker treatment stratification rather than replacement of conventional inflammatory markers; however, whether CCL2 improves prediction beyond CRP, ESR, and clinical disease-activity measures has not been established.

**Figure 3:**
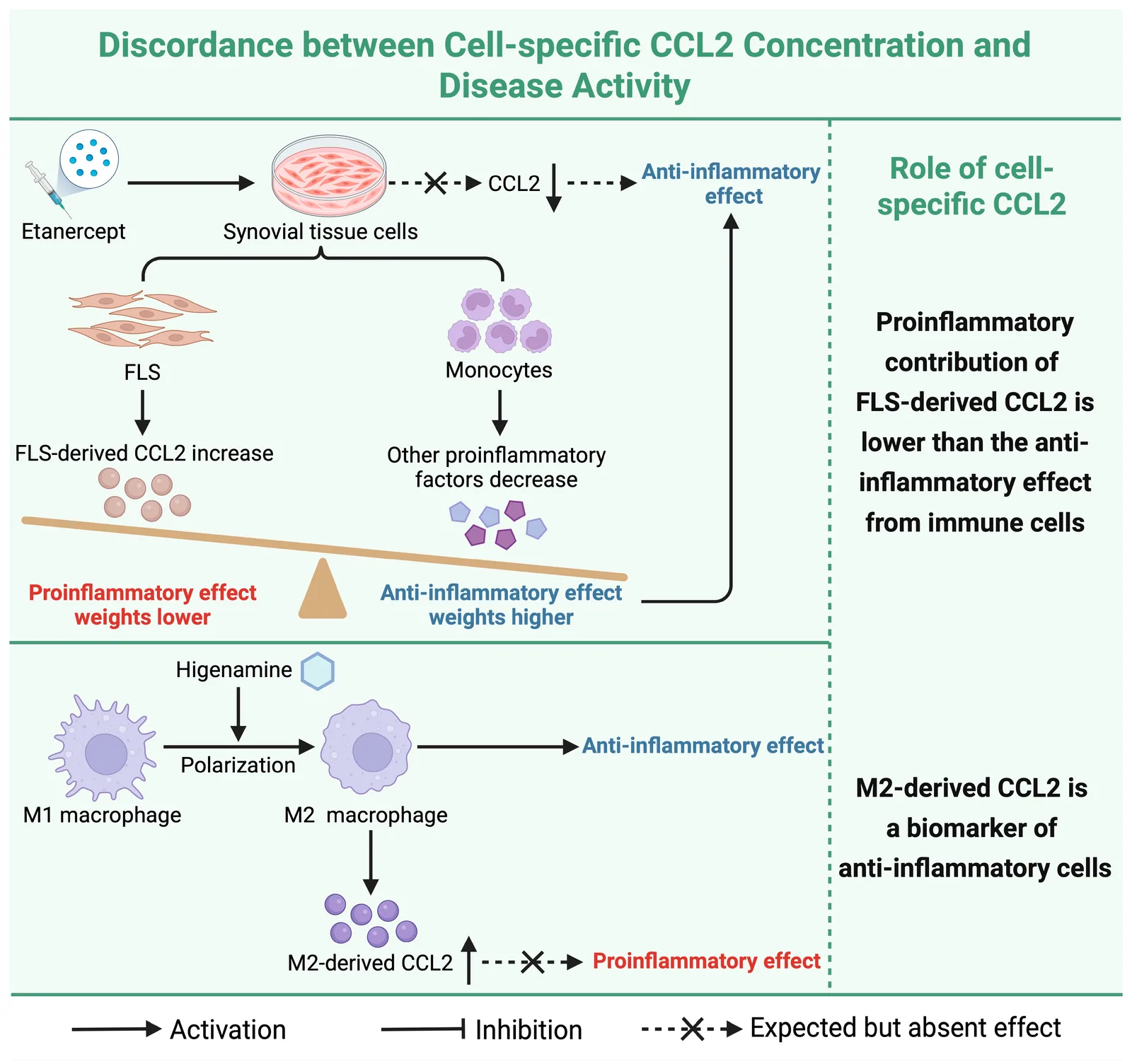
Context-dependent discordance between circulating CCL2 and rheumatoid arthritis disease activity. Top panel: In patients with active RA receiving add-on Etanercept for one year, serum CCL2 increased despite reductions in disease activity, CRP, and ESR. A reason for this is that the anti-inflammatory effect weights higher than proinflammatory effect. Bottom panel: In macrophage-polarization assays and CIA mice, higenamine promoted M1-to-M2 polarization, accompanied by increased CCL2 expression in M2 macrophages and reduced arthritis severity. These observations indicate that changes in CCL2 may reflect treatment-specific, metabolic, and cellular processes and should be interpreted together with conventional clinical and inflammatory indicators.

CCL2 does not always change in parallel with conventional measures of inflammatory activity. In a human observational study, serum CCL2 increased during one year of Etanercept treatment despite improvements in disease activity, CRP, and ESR, and the increase was associated with body weight and BMI [157]. Experimental arthritis provides a second context-dependent example: higenamine promoted M1-to-M2 macrophage polarization while increasing CCL2 expression in M2 macrophages and reducing arthritis severity [182]. These findings indicate that serial CCL2 measurements should be interpreted in relation to treatment exposure, metabolic changes, and cellular source. These mechanisms are discussed further in the Discussion and summarized schematically in Figure 3.

Taken together, the available studies support potential roles for circulating CCL2 in preclinical risk assessment, remission classification, treatment stratification, longitudinal monitoring, and evaluation of selected RA-related complications [15,16,173,183]. Its principal value may lie in complementing conventional inflammatory markers and contributing to multimarker models rather than replacing CRP or ESR. By contrast, the performance and limitations of CRP- and ESR-based disease-activity measures have been characterized across substantially larger and more diverse RA populations [174,175,176,177]. However, reported CCL2 concentrations vary substantially among serum, plasma, and synovial-fluid studies and may be affected by sample type, assay platform, disease stage, treatment exposure, and patient characteristics. Most proposed thresholds and prediction models were derived from individual cohorts and have not been consistently validated in independent populations [173,183]. No standardized RA-specific assay, reference range, or concentration cut-off currently supports routine clinical interpretation. More importantly, it remains unknown whether adding CCL2 to models containing CRP, ESR, and validated clinical disease-activity measures provides reproducible incremental value or improves treatment-related decision making. Future multicenter studies should therefore use standardized sampling and assay procedures, independent validation cohorts, and head-to-head models with and without CCL2 before circulating CCL2 can be considered more than an exploratory complementary biomarker.

## 5. Discussion

CCL2 is not simply an indicator of inflammatory intensity; rather, its biomarker value appears to vary across disease stages. Epidemiologic evidence suggests that CCL2 may participate in early inflammatory processes, particularly in ACPA-positive individuals.

Wide discrepancies in circulating CCL2 concentrations have been reported across RA cohorts. In one longitudinal cohort of 178 patients with RA, the median serum CCL2 concentration was 101.8 pg/mL (interquartile range, 73.6–140.1 pg/mL), and the upper reference limit in healthy controls, defined as the mean plus two standard deviations, was 103.7 pg/mL [173]. By contrast, another study of 111 patients with RA reported a lower median plasma CCL2 concentration of 58.63 pg/mL, with a range of 10.72–1090.16 pg/mL and an interquartile range of 31.5–160.35 pg/mL [172]. Direct comparison between these studies is difficult because serum and plasma measurements are not interchangeable. CCL2 concentrations may also vary according to the detection platform, disease activity, treatment regimen, and individual patient characteristics. In addition, substantial overlap between patients with RA and healthy controls has been observed across studies. Accordingly, no validated serum CCL2 cut-off is currently available for routine diagnosis or disease-activity monitoring. In clinical practice, serum CCL2 should therefore be interpreted together with physical examination findings, CRP, ESR, and validated composite activity scores rather than as a stand-alone biomarker.

Pathologically, vascular proliferation and synovial hyperplasia are the predominant features of RA in the early stage [184], which may present without apparent symptoms [185]. CCL2 not only promotes FLS proliferation but also stimulates VEGF production through the PI3K/p38 pathway, thereby contributing to vascular and synovial hyperplasia [56].

In advanced RA, CCL2 may be less reflective of acute inflammatory intensity and more involved in sustaining chronic inflammation within the inflammatory network. In an AIA model, CCL2 remained elevated beyond 2 weeks, whereas other proinflammatory mediators increased earlier, suggesting that CCL2 may not primarily reflect the acute inflammatory response [186]. Advanced RA is characterized by bone erosion and systemic complications [187]. In animal studies, CCL2 promoted the expansion of OCPs and their recruitment to inflamed joints [74]. Common RA complications include vasculopathy, RA-ILD, and chronic pain. In experimental models, CCL2 contributed to aortic endothelial dysfunction through MCP-induced protein [78]. In addition, α(1,2)-linked fucosylated CCL2 enhanced endothelial-cell migration and tube formation, suggesting a potential association with cardiovascular risk [17]. 

CCL2 has been investigated as a candidate adjunctive biomarker for RA-ILD. In a nested observational study comprising 35 patients with RA-ILD and 35 patients with RA without ILD, higher circulating CCL2 levels were associated with the presence of RA-ILD, whereas CCL2 was not identified as a predictor of subsequent ILD progression [188]. In another single-centre cohort comprising 21 patients with RA-ILD, 25 patients with RA without ILD, and 21 patients with idiopathic pulmonary fibrosis (IPF), serum CCL2 yielded an area under the receiver operating characteristic curve of 0.765 for distinguishing RA-ILD from RA without ILD and 0.863 for distinguishing RA-ILD from IPF [16]. However, given the small sample sizes, single-centre designs, and lack of independent validation, CCL2 should currently be regarded as a candidate component of a multimarker panel rather than a validated stand-alone diagnostic or differential-diagnostic marker. 

Direct clinical evidence regarding longitudinal changes in circulating CCL2 after antifibrotic treatment in patients with RA-ILD remains unavailable. In the RA-ILD subgroup of the INBUILD trial, nintedanib slowed the annual decline in forced vital capacity over 52 weeks compared with placebo, but circulating CCL2 was not included among the reported treatment-response endpoints [158]. A mechanistic study using human monocyte-derived macrophages showed that nintedanib inhibited colony-stimulating factor-1 receptor signalling and reduced CCL2 production; however, this represents in vitro rather than patient-level evidence [159]. Similarly, the TRAIL1 trial evaluated pirfenidone in patients with RA-ILD and reported a slower decline in forced vital capacity, but did not report serial circulating CCL2 measurements [160]. In bleomycin-induced pulmonary fibrosis in mice, pirfenidone reduced pulmonary CCL2 and CCL12 production and inhibited fibrocyte accumulation and migration towards CCL2 [161]. Therefore, current evidence does not establish that nintedanib or pirfenidone alters circulating CCL2 levels in patients with RA-ILD.

Although direct evidence from pulmonary fibroblasts derived from patients with RA-ILD is lacking, in vitro studies have shown that CCL2 can upregulate TGF-β and its receptors in pulmonary fibroblasts. Consequently, TGF-β induces fibrotic phenotypic transformation [189]. Furthermore, CCL2 contributes to the chronic pain of RA. In cells responsive to pain signals, CCR2 and CCL2 are elevated [156]. CCL2 triggers calcium influx in dorsal root ganglia [190]. In addition, CCR2 drives the transport of substance P peripherally [190], and substance P is acknowledged to be associated with pain severity in RA [191]. As for the role in sustaining chronic inflammation, we propose a possible mechanism by which CCL2 sustains chronic inflammation. As abundant endogenous ligands of TLR4 exist in inflamed joints in RA [192], the consistent activation of TLR4 induces macrophage-derived IFN-β, followed by continuous transcription and high expression of macrophage-derived CCL2 via IFN-β-mediated JAK/STAT1 pathways [94].

Overall, the available evidence supports CCL2 as an amplifier of synovial inflammation, although the consistency of this conclusion varies among cell types. Proinflammatory effects in RA-FLS, monocytes/macrophages, and osteoclast-lineage cells have been reported in several experimental settings, whereas the regulatory effects attributed to BMSCs and endothelial-lineage cells are supported by fewer and more model-specific studies [76,88]. The apparent differences may arise from the cellular source of CCL2, the relative expression of CCR2A and CCR2B, the stage of arthritis, and the composition of the local synovial environment [92,93]. These variables make it difficult to combine all cell-specific observations into a single linear pathway. The cell-specific network should therefore be viewed as a context-dependent framework rather than a uniform mechanism operating across all cell populations and stages of RA.

A possible association between cell-specific CCL2 production and ACPA-positive RA has also been suggested, although the available evidence remains indirect. Citrullinated fibrinogen increases CCL2 expression in BMSCs through TLR4–NF-κB signaling [80], while sustained TLR4 activation can maintain macrophage CCL2 production through an IFN-β–JAK/STAT1 feedback loop [94]. Because these findings were obtained from separate experimental settings, they do not yet establish a direct causal relationship between ACPA and persistent CCL2 expression.

RA is more prevalent in women, but current evidence does not demonstrate a clear association between sex and CCL2. At the genetic level, the CCL2 variants rs1024611 G, rs13900 T, and rs4586 C were associated with lower RA risk in women, whereas no similar association was observed in men [193]. Evidence that CCL2 variants increase RA susceptibility specifically in women remains limited. At the protein level, direct evidence linking CCL2 to sex differences in RA is also lacking; the reported absence of an association between DAS28-CCL2 and sex provides only indirect support [172]. Estrogen may exert bidirectional effects on CCL2. It can locally downregulate CCL2, possibly through inhibition of the p38 MAPK/NF-κB pathway [194,195], but may also facilitate neutrophil infiltration [194], and infiltrating neutrophils can increase CCL2 expression in RA synovium [196]. These opposing effects may partly explain the lack of a consistent association between sex and CCL2. Furthermore, TLR2 agonists can activate estrogen-receptor signaling and modulate CCL2 expression [197], suggesting that estrogen-receptor activation may influence CCL2 under endogenous stimulation. Two large clinical trials did not report CCL2 concentrations before and after treatment, but neither demonstrated clinical improvement in RA with estrogen-based therapy [198,199]. Thus, current clinical evidence does not support a clear therapeutic benefit of estrogen in RA.

Direct blockade of the CCL2/CCR2 axis has revealed a clear gap between preclinical target validity and clinical efficacy. Preventive CCL2/CCR2 blockade reduced osteoclast-progenitor homing and bone damage in experimental arthritis [74], whereas a randomized trial of CCR2 modulation produced peripheral biological effects without consistent improvement in synovial inflammation [10]. The ABN912 trial likewise failed to improve clinical or synovial outcomes and caused a marked increase in measured total CCL2, consistent with the delayed clearance of antibody–CCL2 complexes [96]. Because total CCL2 includes antibody-bound ligand, this increase should not be interpreted as evidence that free, biologically active CCL2 increased or as the sole explanation for treatment failure.

The translational gap is more plausibly explained by the broader chemokine network and disease context. CCR2 has multiple ligands, and inflammatory cells use overlapping chemokine receptors, allowing alternative recruitment pathways to remain active after isolated CCL2/CCR2 blockade [95]. Pharmacokinetic properties may provide an additional limitation. A structure–kinetic study of cyclopentylamine CCR2 antagonists reported a receptor residence time of approximately 2.4 min for one tested compound [200]. This relatively short residence time may make sustained target blockade difficult to achieve in chronic disease. Treatment timing may also matter: the favourable experimental study used preventive intervention before persistent synovial circuits were established [74], whereas the CCR2 clinical trial involved patients with established RA [10]. In established disease, monocyte recruitment is only one component of a broader synovial network, and cell-specific differences between CCR2A and CCR2B may add further variability to target engagement and signalling [92,93].

By contrast, JAK inhibitors are established therapies for RA [133], and Baricitinib suppresses CCL2 together with IL-6 and other proinflammatory mediators in ex vivo synovial-derived cells [135]. This comparison supports network-level modulation rather than isolated ligand–receptor blockade, although a reduction in CCL2 should be interpreted as one component of the therapeutic response rather than proof that CCL2 is its principal mediator. Overall, CCL2 remains a biologically relevant node and a potential component of stratified or combination strategies, but current evidence does not support direct CCL2/CCR2 blockade as a broadly effective stand-alone treatment for RA. Taken together, the current translational evidence supports CCL2 more strongly as an exploratory complementary biomarker than as a stand-alone therapeutic target. Treatment-related changes in CCL2 are better interpreted as one component of broader pathway modulation rather than as a primary mechanism of therapeutic efficacy.

**Figure 4:**
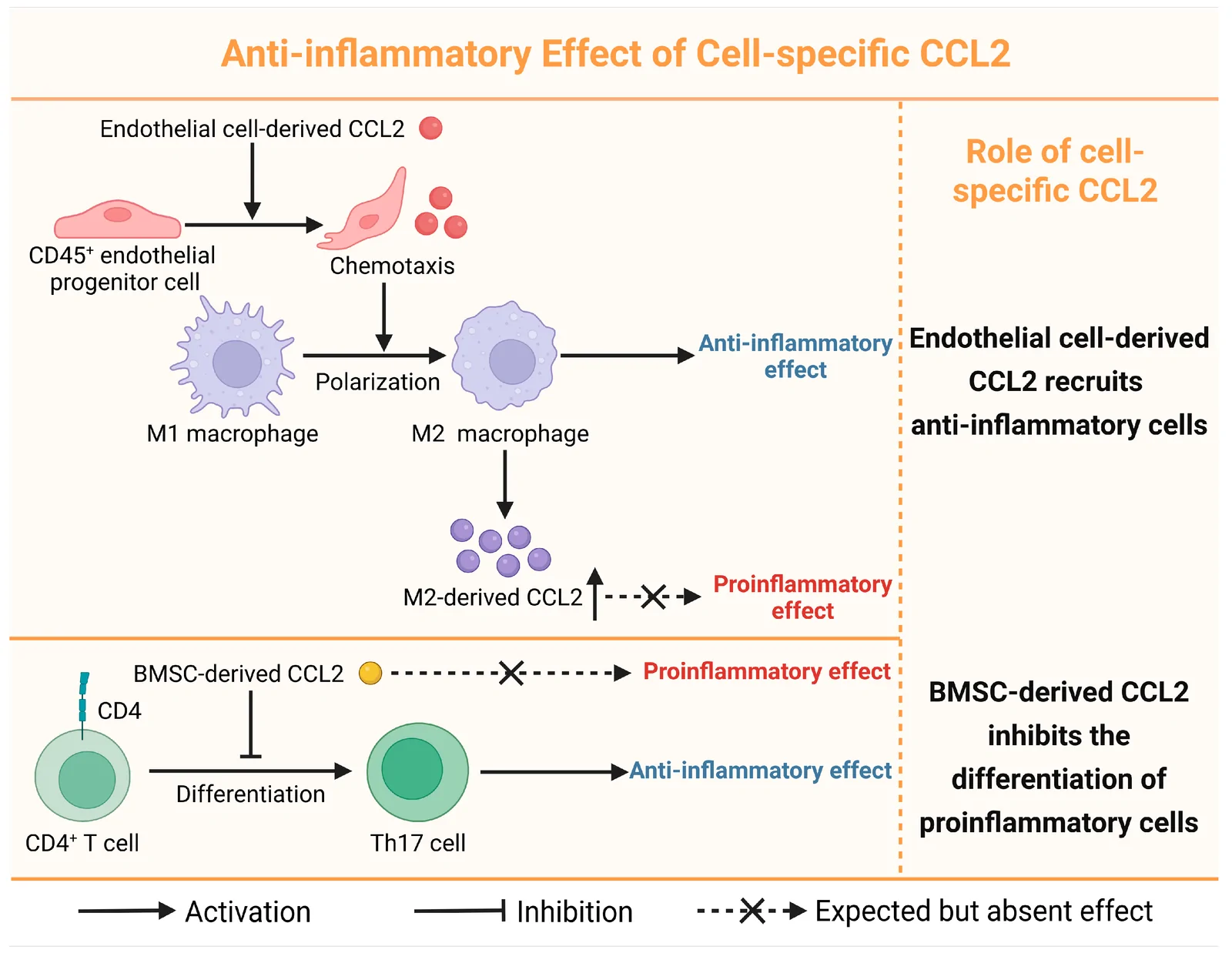
Anti-inflammatory effect of cell-specific CCL2. Top panel: Endothelial cell-derived CCL2 recruits CD45^+^ endothelial progenitor cells to induce M2-type polarization of macrophages. Bottom panel: BMSC-derived CCL2 exerts an anti-inflammatory effect by inhibiting differentiation of Th17 cells.

Huang et al. proposed that the increase in CCL2 after Etanercept treatment may be related to higher BMI. However, the relationship between RA status and BMI remains uncertain [201]. In female cynomolgus monkeys with experimental RA, CCL2 showed a moderate inverse correlation with body weight, which contrasted with the expected positive relationship [202]. Thus, BMI alone may not fully explain the post-treatment increase in CCL2. Moreover, CCL2 does not consistently decrease after Etanercept exposure. An in vitro study reported that 48 h of Etanercept treatment failed to reduce CCL2 in cultures containing FLS and peripheral blood mononuclear cells [203] (Figure 4). One possible explanation is that CCL2 suppression may be weaker in FLS-predominant than in immune-cell-predominant cultures; however, this interpretation remains speculative. The study by Huang et al. included only 40 Etanercept-treated patients from a single city. Therefore, cohort-specific cellular or metabolic factors cannot be excluded. 

**Figure 5:**
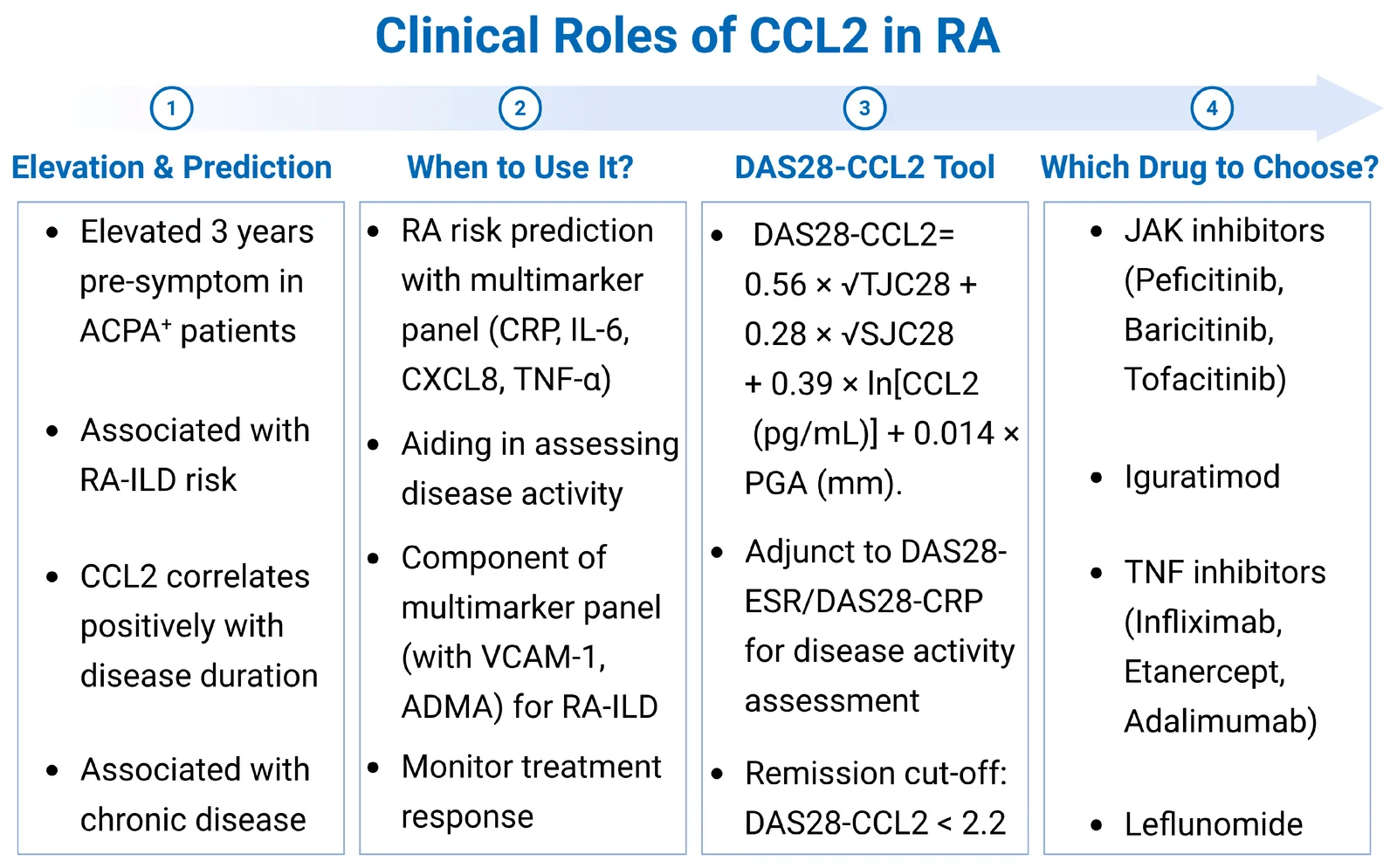
Clinical roles of CCL2 in RA. Panel 1: CCL2 elevation contexts and predictive associations. Serum CCL2: median 101.8 pg/mL (IQR 73.6–140.1); plasma CCL2: median 58.63 pg/mL (IQR 31.5–160.35). Serum and plasma values are not interchangeable. Panel 2: clinical use recommendations. However, standardized assays and validated cut-offs are still lacking for routine use. Panel 3: DAS28-CCL2 scoring system and remission cut-off (<2.2). Panel 4: CCL2-reducing drugs. Among different drug categories, JAK inhibitors seem to decrease CCL2 most.

In summary, we illustrate the clinical role of CCL2 in RA (Figure 5), including conditions associated with elevated CCL2, its potential predictive applications, the DAS28-CCL2 score, and therapeutic approaches reported to alter CCL2 levels.

## 6. Future Directions

Current evidence supports CCL2 as a biologically relevant but context-dependent component of RA, and several questions remain unresolved. Single-cell RNA sequencing and spatial transcriptomics could identify synovial cell subsets that produce or respond to CCL2 at different disease stages. These approaches could also clarify how local tissue niches and CCR2 expression shape the effects of CCL2 [76,88,92,93]. Future studies should also determine whether sex modifies circulating or tissue CCL2 patterns and whether cell-specific post-translational modifications alter CCL2 activity [17,193,201,204]. Paired blood and synovial sampling before and after treatment would help establish when circulating CCL2 reflects local joint inflammation [10,96].

Clinical translation will require patient stratification rather than further evaluation in unselected RA populations. Multicentre studies should assess CCL2 within standardized multimarker panels that include established inflammatory markers and other chemokines, followed by independent validation across disease stages and treatment groups [172,173,178,183]. Artificial intelligence-assisted analysis may support biomarker selection and the identification of CCL2-related molecular subgroups, although model performance must be confirmed in external cohorts. The redundancy of the chemokine network also supports the investigation of rational combinations targeting CCL2/CCR2 together with complementary chemokine or cytokine pathways [95,133,135]. Such studies should incorporate evidence of synovial target engagement and molecularly defined patient subgroups rather than relying on circulating CCL2 alone [10,96].

## 7. Conclusions

CCL2 is best viewed as a context-dependent cross-cellular mediator within the broader inflammatory network of RA. Experimental studies support its biological involvement in leukocyte recruitment, synovial fibroblast responses, and osteoclast-related bone remodeling, whereas human evidence remains predominantly associated. Its effects are shaped by cellular source, CCR2 expression, disease stage, and the local tissue environment. These context-dependent factors may help explain why substantial preclinical evidence has not translated into consistent clinical benefit from direct CCL2/CCR2 blockade [74,92,93,96]. Circulating CCL2 may provide complementary information on disease activity and treatment outcome, particularly within multimarker models, although no standardized assay or validated cutoff currently supports routine clinical use [172,173,178,183]. CCL2 should therefore be regarded as part of a broader inflammatory network rather than as a validated stand-alone therapeutic target or biomarker.

## Figures and Tables

**Table 1:** Upstream molecular regulators of CCL2 expression in rheumatoid arthritis.

Regulatory Class	Regulator(s)	Principal Cell/Model	Effect on CCL2 and Major Pathway	References
p38 MAPK/NFAT5	IL-1β, TGF-β, IL-18	RA-FLS and endothelial cells	IL-1β/TGF-β increases CCL2 through p38 MAPK–NFAT5. IL-18 activates p38-linked endothelial inflammatory signaling.	[6,23]
NF-κB and STAT3	TNF-α, IL-6, IL-7, IL-33	Synovial cells and RA-FLS	TNF-α activates ROS–NF-κB; IL-6 amplifies TNF-α and forms a STAT3–NF-κB complex with IL-7. TNF-α and IL-33 form a p38-dependent amplification loop.	[24,25,26,27,28]
PI3K/AKT and ERK/JNK	IL-17; early macrophage activation	RA-FLS and macrophages	IL-17 induces CCL2 through PI3K/ERK/JNK in RA-FLS and PI3K/ERK in macrophages. Early macrophage production may involve PI3K/AKT and MEK/ERK1/2.	[13,29]
Indirect and synergistic cytokine circuits	Oncostatin M + IL-1α; IL-22 and IL-1β; IL-1β/IL-6 → CCL25	RA-FLS, antigen-presenting cells, and macrophages	Oncostatin M and IL-1α synergistically increase CCL2. IL-22 may act indirectly through IL-1β. CCL25 promotes a non-classical M1 phenotype that produces CCL2 and CXCL8.	[30,31,32,33,34]
Context-dependent cytokines	IFN-γ + IL-15; IFN-γ; IL-4; IL-4 + IL-15	RA-FLS	IFN-γ/IL-15 increases CCL2, whereas IFN-γ can suppress CCL2 in other settings. IL-4 may increase basal CCL2 but inhibit IL-15-induced CCL2.	[18,21,22]
Adipokine/metabolic regulation	Visfatin/PBEF, adiponectin, nesfatin-1, lipocalin-2; reduced PPARγ	RA-FLS, chondrocytes, macrophages, synovium, and white adipose tissue	These factors generally reinforce CCL2-associated inflammation and M1 polarization. Lipocalin-2 evidence is correlative; reduced PPARγ accompanies the inflammatory adipose-tissue profile.	[19,35,36,37,38,39]
miRNA-mediated derepression	miR-124a/circRNA-HIPK3; miR-23a/IKKα; TNF-α/IL-1β–NF-κB–YY1–miR-10a	RA synovial cells, articular cartilage, and RA-FLS	miR-124a directly suppresses CCL2 mRNA, whereas circRNA-HIPK3 reduces miR-124a activity. Loss of miR-23a derepresses IKKα, and TNF-α/IL-1β-induced YY1 suppresses miR-10a; both routes enhance CCL2 expression.	[20,40,41,42]

**Table 3:** Categories and mechanisms of artificial synthetic compounds targeting CCL2 in RA. Evidence levels are abbreviated as follows: RCT, randomized controlled trial. “Failed” denotes a negative outcome reported in the cited study. Animal doses, in vitro concentrations, and clinical regimens are presented according to the original studies. Values reported for negative studies indicate tested rather than effective doses. i.p., intraperitoneal; p.o., oral; i.v., intravenous; s.c., subcutaneous; ED50, median effective dose.

Subcategory	Compound	Dose/Regimen	Mechanism	Evidence/Status	Main Limitation	References
CCL2/CCR2 Axis-Targeting	RS504393 (CCR2 antagonist)	4 mg/kg, i.p., every 48 h until the experimental endpoint	Blockade of CCR2; improvement of bone erosion	Animal model; preclinical	Preventive treatment in a single animal model	[74]
	MK0812 (CCR2 antagonist)	30 mg/kg, p.o., b.i.d.	Blockade of CCR2	Animal model; negative	Limited efficacy in the cited arthritis model	[155]
	ABN912 (CCL2 monoclonal antibody)	0.3, 1, 3, or 10 mg/kg, i.v. infusion on days 1 and 15	Neutralization of CCL2	Phase II RCT; negative	No clinical or synovial improvement; total CCL2 increased after treatment	[96]
	CNTX-6970 (CCR2 and CCR5 antagonist)	100 or 300 mg, p.o., b.i.d. for 6 weeks	Blockade of CCR2	Phase II RCT; evaluated outside RA	No direct evidence for RA	[156]
	INCB3344 (CCR2 antagonist)	5 or 10 nM in vitro; 10 mg/kg, i.p., once every 3 days from day 29 in CIA mice	Blockade of CCR2; improvement of bone erosion	In vitro + animal; preclinical	No clinical validation; uncertain translation to established RA	[126]
Conventional DMARDs	Combination of MTX and carnosic acid or N-feruloylserotonin	Carnosic acid: 100 mg/kg/day, p.o.; MTX: 0.3 mg/kg, p.o., twice weekly N-feruloylserotonin: 15 mg/kg/day, p.o.; MTX: 0.3 mg/kg, p.o., twice weekly	Inhibition of NF-κB pathway; potentiating the therapeutic effect of MTX low dose	Animal model; preclinical combination strategy	Combination effect cannot be attributed specifically to CCL2 or one component	[129,130]
	Galantamine	1.25, 2.5, or 5 mg/kg/day, p.o., for 2 weeks	Upregulation of acetylcholine concentration to inhibit cytokine production and macrophage activation	Animal model; repurposing concept	Animal evidence only; indirect effect on CCL2	[132]
	Leflunomide	100 mg/day, p.o., for 3 days, followed by 20 mg/day for 12 months	Downregulation of CCL2	Human observational study; established RA therapy	Observational design; no evidence that CCL2 reduction mediates clinical benefit	[131]
Kinase Inhibitors & Transcriptional Modulators	BTK inhibitor PCI-32765	Oral ED50: 2.6 mg/kg/day; 12.5 mg/kg/day, p.o., for 18 days in mechanistic analyses	Inhibition of monocyte and macrophage infiltration	Animal model; preclinical	Broad BTK effects; CCL2 was a secondary endpoint	[139]
	CX-4945 (Silmitasertib)	2.5 or 5 μM in vitro; 75 mg/kg/day, i.p., on days 18–46 in CIA mice	Inhibition of casein kinase 2 to inhibit RA-FLS proliferation	In vitro + animal; preclinical	Broad CK2 effects; no clinical RA validation	[140]
	KRN5	15 or 60 mg/kg, p.o., every other day for 3 weeks, beginning on day 21	Downregulation of NFAT5; inhibition of the NF-κB pathway	Animal model; preclinical	Single animal study; no human target-engagement data	[141]
	Iguratimod	25 mg/day for the first 4 weeks, followed by 50 mg/day for 24 weeks	Inhibition of JNK/p38 signaling and suppression of CCL2 expression, proliferation, migration, and invasion in RA-FLS	Phase III RCT + separate in vitro mechanistic study; clinically established evidence in active RA	CCL2 was not measured clinically; efficacy cannot be attributed to CCL2 suppression	[136,137]
Receptor-Targeting Therapies	All-trans retinoic acid (RAR ligand)	0.5 mg/mouse, i.p., three times weekly for 35 days	Inhibition of macrophage infiltration; downregulation of CCL2	Animal model; preclinical	Pleiotropic receptor effects; animal evidence only	[142]
	JWH133/JWH-015 (CB2 agonists)	JWH133: 1 or 4 mg/kg/day, i.p., divided into two daily injections for 21 days	Activation of cannabinoid receptor 2 to downregulate FLS-derived CCL2	In vitro + animal; preclinical	Results derive from separate studies and experimental systems	[143,144]
	Compound 32 (GPR183 antagonist)	0.1, 0.3, or 1.0 mg/kg, p.o., twice daily from day 21; minimum efficacious dose: 0.1 mg/kg	Downregulation of MMPs, CCL2, and VEGF	In vitro + animal; preclinical	No clinical validation; effects are not limited to CCL2	[145]
Anti-TNF Therapy	Infliximab	3 mg/kg, i.v., at baseline and weeks 2 and 6, then every 8 weeks	Inhibition of the effect of TNF	Human observational studies; established RA therapy	No causal evidence that CCL2 changes mediate clinical response	[146,148,149]
	Anti-TWEAK mab	250 μg/mouse, i.p., once daily for 2 weeks from day 21	Inhibition of inflammatory cells infiltration; downregulation of CCL2 and MIP-2	In vitro + animal; preclinical	No human efficacy data; CCL2 is one of several mediators	[151]
	Etanercept	25 mg, s.c., twice weekly for at least 1 year	Inhibition of TNF	Human observational study; established RA therapy	CCL2 increased despite improvement in disease activity	[157]
	Anti-CD11b mAb	200 μg/mouse, i.p., twice weekly for 6 months	Inhibition of active B cells; inhibition of the infiltration of osteoclasts and inflammatory cells	Animal model; preclinical	Animal study only; no human validation	[150]
Chemical Compound	P-Coumaric acid	100 mg/kg, i.p., once daily for 16 days (days 11–26)	Inhibition of NF-κB pathway; downregulation of proinflammatory cytokines	In vitro + animal	Broad anti-inflammatory effects; direct target unclear	[152]
	BT2	3 or 30 mg/kg, i.p., administered on day 3 together with LPS	Inhibition of the ERK pathway	In vitro + animal; preclinical	Acute experimental model; no human efficacy data	[153]
Antifibrotic therapy for RA-ILD	Nintedanib	150 mg, p.o., b.i.d. in the INBUILD trial; 0.01–1 μM in human monocyte-derived macrophages	Slowed FVC decline in progressive RA-ILD; inhibited CSF1R signalling and reduced macrophage CCL2 production in vitro	Randomized trial subgroup + human in vitro study; clinically evaluated in RA-ILD	No longitudinal patient CCL2 data; CCL2 modulation supported by in vitro evidence	[158,159]
	Pirfenidone	2403 mg/day, p.o., in TRAIL1; 300 mg/kg/day, p.o., for 2 weeks in bleomycin-treated mice	Slowed FVC decline in RA-ILD; reduced pulmonary CCL2/CCL12 production and fibrocyte accumulation and migration in mice	Phase II trial + animal/in vitro study; clinically evaluated in RA-ILD	No longitudinal patient CCL2 data; modulation based on experimental fibrosis models	[160,161]

**Table 4:** Categories and mechanisms of novel therapies targeting CCL2 in RA. All studies summarized in this table were conducted in animal models. These approaches remain preclinical, and none of the cited studies establishes clinical efficacy or routine use in patients with RA. The principal limitations listed here are based on the experimental design and translational scope of the cited studies.

Subcategory	Compound	Mechanism	Main Limitation	References
Mesenchymal Stem Cell Therapy	Combination of MSCs and IL-4	Downregulation of TNF-α, CCL2, MMP-1	CCL2-specific contribution remains unclear	[162]
Gene Therapy	SIRT6 adenoviral delivery	MyD88-ERK pathway inhibition	Clinical safety, durability, and immunogenicity unassessed	[166]
Nanoparticle-Based Therapy	Piroxicam nanoparticles	Inhibition of NF-κB pathway	Effects of piroxicam and the nanoparticle carrier were not fully separated	[167]
Nanoparticle-Based Therapy	Selenium nanoparticles	Downregulation of CCL2	Direct target and long-term safety unclear	[168]
Nanoparticle-Based Therapy	Eugenol-chitosan nanoparticles	Downregulation of CCL2 and TGF-β	Eugenol and carrier effects not separated	[169]
Nanoparticle-Based Therapy	Meuktx-chitosan nanoparticles	Downregulation of CCL2, TGF-β and caspase-8	Delivery stability and long-term safety untested clinically	[170]
Light Therapy	LED irradiation	Inhibition of CCL2 gene expression	Single animal study; parameters not standardized	[164]
Light Therapy	Low-level laser irradiation	Downregulation of CCR2 mRNA	Single animal study; protocol not standardized	[165]

## Data Availability

No new data were created or analyzed in this study. Data sharing is not applicable to this article.

## Supplementary Materials

The following supporting information can be downloaded at: https://www.mdpi.com/article/10.3390/cells15161461/s1, Table S1: In vitro studies on CCL2-centered therapies [49,71,134,135,138,162,163,203,205,206,207,208,209,210,211,212,213,214,215,216,217,218,219,220].

## Abbreviations

The following abbreviations are used in this manuscript:

ACPA	Anti-citrullinated protein antibody
AIA	Antigen-induced arthritis
BMI	Body mass index
BMSC	Bone marrow mesenchymal stem cell
BTK	Bruton’s tyrosine kinase
CB2	Cannabinoid receptor 2
CCL2	C-C motif chemokine ligand 2
CCR2	C-C chemokine receptor type 2
CIA	Collagen-induced arthritis
CK2	Casein kinase 2
CREB	cAMP response element-binding protein
CRP	C-reactive protein
DAS28	Disease activity score in 28 joints
DMARDs	Disease-modifying antirheumatic drugs
EGF	Epidermal growth factor
ERK	Extracellular signal-regulated kinase
ESR	Erythrocyte sedimentation rate
FLS	Fibroblast-like synoviocyte
FNDC4	Fibronectin type III domain-containing protein 4
GPR	G-protein receptors
IKKα	IκB kinase alpha
JAK	Janus kinase
JNK	C-Jun NH2-terminal kinase
LED	Light-emitting diode
LPA	Lysophosphatidic acid
LPAR1	Lysophosphatidic acid receptor 1
LPS	Lipopolysaccharide
LTB4	Leukotriene B4
MAPK	Mitogen-activated protein kinase
MCP-1	Monocyte chemoattractant protein-1 (historical name for CCL2)
MyD88	Myeloid differentiation primary response 88
miRNA	MicroRNA
MMP-9	Matrix metalloproteinase 9
MSC	Mesenchymal stem cell
MTX	Methotrexate
NF-κB	Nuclear factor κB
NFAT5	Nuclear factor of activated T cells 5
OCP	Osteoclast precursor
OPN	Osteopontin
PI3K	Phosphoinositide 3-kinase
RA	Rheumatoid arthritis
RA-FLS	Fibroblast-like synoviocyte from Rheumatoid arthritis patients
RA-ILD	RA-associated interstitial lung disease
RAR	Retinoic acid receptor
RCT	Randomized controlled trial
ROS	Reactive oxygen species
SF	Synovial fluid
SIRT6	Sirtuin 6
ST	Synovial tissue
STAT	Signal transducer and activator of transcription
TLR4	Toll-like receptor 4
TNF	Tumor necrosis factor
TWEAK	TNF-like weak inducer of apoptosis
VCAM-1	Vascular cell adhesion molecule-1
VEGF	Vascular endothelial growth factor
