# Supplementary material for: CCL2 in Rheumatoid Arthritis: A Context-Dependent Cross-Cellular Node Serving as Biomarker and Therapeutic Target
Source: Cells. 2026 Aug 14;15(16):1461. doi: 10.3390/cells15161461 (PMC13510963; doi:10.3390/cells15161461)
Supplement: Supplementary file 1 [file cells-15-01461-s001.zip › cells-4418189-supplementary.pdf]

Table S1. In vitro studies on CCL2-centered therapies. All studies summarized in this table were conducted in vitro. Human clinical study indicates that the intervention has been evaluated in patients with RA and does not necessarily indicate direct targeting of CCL2. A negative result was reported in the cited study.

Subcategory	Compound	Mechanism	Development stage/model	References
Herb Monomer	Columbianadin	Inhibition of dendritic cells maturity induced by TNF-α	In vitro only	[205]
	Honokiol	Inhibition of NF-κB pathway	In vitro and animal model	[206]
	Resveratrol	Downregulation of CCL2 by blocking the TLR4-mediated NF-κB pathway; combination of resveratrol and MTX enhances therapeutic effect	In vitro and ex vivo	[207,208]
	Salvianolic acid B	Upregulation of miR-142-3p to inhibit NF-κB and JNK pathways	In vitro only	[209]
	Cantleyoside	Inhibition of RA-FLS proliferation; regulation of AMPK/Sirt 1/NF-κB pathway	In vitro only	[210]
	Stauntoside B	Inhibition of CCL2, IL-1β, TNF-α, IL-6, PGE_2, NO release in macrophages	In vitro only	[211]

	Peficitinib	Regulation of STAT1, STAT3, and STAT5; inhibition of CCL2 secretion, monocyte migration, and RA-FLS proliferation	In vitro and human clinical study	[134]
	Baricitinib	Blockade of JAK signaling from oncostatin M in FLS; downregulation of IL-6 and CCL2	Ex vivo and human clinical study	[135]
Kinase Inhibitors & Transcriptional Modulators	PF-956980*	Inhibition of JAK3	In vitro only	[138]
	INCB028050	Inhibition of JAK-1/-2/-3	In vitro and human clinical study	[138]
	15d-PGJ2 or ciglitazone	Activation of PPARγ; inhibition of osteoclasts proliferation; inhibition of the NF-κB pathway	In vitro only	[71]
	Secreted frizzled-related protein 5	Inhibition of the JNK pathway; competing for Wnt binding sites	In vitro only	[212]
Receptor-Targeting Therapies	Bexarotene (RXR ligand)	Inhibition of p38 MAPK and NF-κB pathway	In vitro only for RA	[213]
	VIP	Downregulation of FLS-derived CCL2 induced by the activation of	In vitro only	[49]

		TLR4		
	GPR43 agonist	Inhibition of NF-κB pathway	In vitro only	[214]
	TC-G1008 (GPR39 agonist)	Inhibition of NF-κB, JNK pathways	In vitro only	[215]
Chemical Compound	β-D-mannuronic acid	Downregulation of CCL2 mRNA	In vitro and human clinical study	[216]
	2′-methylflavanone or 3′-methylflavanone	Inhibition of macrophage-derived CCL2	In vitro only	[217]
	Phenylpropanoid-loaded exosomes	Inhibition of ERK/MAPK and IκB / NF-κB signaling pathways	In vitro only	[218]
	10-NO_2-OA	Downregulation of CCL2	Ex vivo only	[203]
Mesenchymal Stem Cell Therapy	Co-culture of hUC-MSCs and FLS	Downregulation of CCL2; chondrogenesis promotion	In vitro; related MSC approaches include animal and human studies	[162,163,219]
Gene Therapy	CAV1 knockdown	CCL2 transcriptional downregulation	In vitro only	[220]
